# Autophagy: an adaptive physiological countermeasure to cellular senescence and ischaemia/reperfusion‐associated cardiac arrhythmias

**DOI:** 10.1111/jcmm.13053

**Published:** 2016-12-20

**Authors:** Istvan Lekli, David Donald Haines, Gyorgy Balla, Arpad Tosaki

**Affiliations:** ^1^ Department of PharmacologyFaculty of PharmacyUniversity of DebrecenDebrecenHungary; ^2^ Department of PediatricsMedical and Health Science CenterUniversity of DebrecenDebrecenHungary; ^3^ Hemostasis, Thrombosis and Vascular Biology Research GroupHungarian Academy of SciencesDebrecenHungary

**Keywords:** autophagy, cardiovascular system, proteotoxic stress

## Abstract

Oxidative stress placed on tissues that involved in pathogenesis of a disease activates compensatory metabolic changes, such as DNA damage repair that in turn causes intracellular accumulation of detritus and ‘proteotoxic stress’, leading to emergence of ‘senescent’ cellular phenotypes, which express high levels of inflammatory mediators, resulting in degradation of tissue function. Proteotoxic stress resulting from hyperactive inflammation following reperfusion of ischaemic tissue causes accumulation of proteinaceous debris in cells of the heart in ways that cause potentially fatal arrhythmias, in particular ventricular fibrillation (VF). An adaptive response to VF is occurrence of autophagy, an intracellular bulk degradation of damaged macromolecules and organelles that may restore cellular and tissue homoeostasis, improving chances for recovery. Nevertheless, depending on the type and intensity of stressors and inflammatory responses, autophagy may become pathological, resulting in excessive cell death. The present review examines the multilayered defences that cells have evolved to reduce proteotoxic stress by degradation of potentially toxic material beginning with endoplasmic reticulum‐associated degradation, and the unfolded protein response, which are mechanisms for removal from the endoplasmic reticulum of misfolded proteins, and then progressing through the stages of autophagy, including descriptions of autophagosomes and related vesicular structures which process material for degradation and autophagy‐associated proteins including Beclin‐1 and regulatory complexes. The physiological roles of each mode of proteotoxic defence will be examined along with consideration of how emerging understanding of autophagy, along with a newly discovered regulatory cell type called telocytes, may be used to augment existing strategies for the prevention and management of cardiovascular disease.

## Ischaemia–reperfusion injury and ventricular fibrillation

### Inflammatory tissue damage, disease and trauma

The major symptoms of all diseases known at the time of this writing are due at some level to inflammatory adaptive processes leading to autoreactive tissue damage. Typically, microbial or toxic challenge, physical injury, or disruption of normal physiology by genetic defect or neoplasm triggers defensive responses that have evolved to counteract primary threats, but that may remain activated at high levels, causing prolonged and deleterious pro‐inflammatory signalling. Failure of regulatory mechanisms to restore these processes to normal activity levels results in chronic inflammation, characterized by sustained production of highly reactive oxygen‐containing compounds called reactive oxygen species (ROS) that subject tissue to oxidative stress—often for the life‐time of an individual. Disruption of control over inflammation in cardiovascular tissue disrupts co‐ordinated contractility of heart muscle, resulting in VF and other arrhythmic disorders 1, 2. A primary trigger for the onset of chronic inflammatory syndromes is ischaemia, a condition in which functional impairment and loss of structural integrity of affected tissue occur as a consequence of oxygen and nutrient deprivation 3. These processes cause increased generation of reactive oxygen‐containing molecules by cardiovascular and immune cells, which promote tissue damage and impair cardiac function 1, 4. Interestingly, subjecting the heart to very brief periods of ischaemia protects the myocardium from subsequent ischaemic insult. A major mechanism contributing to this effect has been shown to involve ROS‐mediated induction of antioxidant enzymes, prominently superoxide dismutase, which remain elevated and/or increase in responsiveness, with resulting increases in the efficiency with which ROS are scavenged 5, 6. Recent work by our laboratory has demonstrated that naturally occurring inducers of another major antioxidant defence enzyme, haem oxygenase‐1, also exhibit powerful cardioprotective effects 7, 8.

### Ischaemia‐mediated disruption of cellular and tissue homoeostasis

Ischaemic effects are associated with conditions of disease or trauma affecting circulation and may also occur following surgery in which blood supply to a tissue or organ is intentionally restricted. A resulting hyperactivation of oxidative metabolism occurs as a result of intracellular accumulation of highly reactive oxygen‐containing compounds (reactive oxygen species—ROS) along with increased activity by proteolytic enzymes. The outcome of cellular damage, typically interfere with normal homoeostatic signalling processes (in particular those involving calcium), disrupting mitochondrial activity and causing loss of cell compartmentalization and membrane integrity 9, which in heart tissue, may manifest as arrhythmias 1, 2.

### Ischaemia–reperfusion (IR) injury and arrhythmias

Restoration of blood flow to ischaemic tissue results in high levels of ROS activity and resulting oxidative stress, following restoration of circulation, an effect called ischaemia–reperfusion (IR) injury 9, 10. Such reoxygenation by blood reperfusing ischaemic tissue restores some aspects of healthy homoeostasis; however, rapid increases in ROS levels also trigger abnormally intense inflammation, leading to debilitation and occasionally death when a vital organ is affected 3. Cells of the cardiovascular, kidney and nervous systems are particularly susceptible to IR injury due the stringent requirement of these cells on normal cellular redox balance 11, 12.

A hallmark of IR injury to the heart and a major cause of death in human cardiovascular disease are fibrillation, an arrhythmic condition which develops from severe disruption of cardiac rhythm triggered by chemical and physical damage to cells of the heart 13. A commonly occurring and often fatal form of this syndrome, which affects ventricular myocardium, is VF 1, 14. IR‐induced VF occurs suddenly after the onset of reflow and has been observed in all animal species 15. In humans, this syndrome occurs during thrombolytic therapy and cardiac surgery 16, 17. IR‐induced injury to the myocardium often is observed with cardiovascular pathologies, including heart failure coronary artery lesions and arrhythmias, causing cell death through the major mechanisms including necrosis, apoptosis and autophagy, which can be together termed as ‘necroapoptophagy’ 18.

## Consequences of IR injury and physiological countermeasures

### Proteotoxic stress, cellular senescence and adaptive countermeasures

The major symptoms of cardiovascular disease, including VF, which are often initiated by IR‐associated oxidative damage, also may be exacerbated by hypertrophic cell growth resulting from greatly elevated activation of a serine/threonine kinase responsible for synthesis of cell components called mammalian target of rapamycin (mTOR) 19. Damaged cells with high mTOR activity often lose their proliferative potential, proper control over proteostasis and accumulate aggregates of protein precursors, which disrupt normal cellular metabolism that leads to a phenomenon known as proteotoxic stress 20. Proteotoxic stress‐induced cell enlargement not counterbalanced by proliferation causes emergence of senescent cellular phenotypes, in which build‐up of incorrectly folded polypeptide aggregates disrupts compartmentalization of cell components, stimulating elevated expression of inflammatory of cytokines 18. As an organism ages, each component tissue accumulates steadily increasing numbers of senescent cells, which cause tissue environments to become progressively more pro‐inflammatory 21. Age‐associated increase in senescent cell numbers is augmented by stress placed on tissues as a result of trauma or disease, resulting in oxidative damage‐induced conversion of cells to senescent phenotypes—a process known as stress‐induced premature senescence (SIPS) 21. Progressively, pro‐inflammatory tissue environments resulting from build‐up of senescent cells promote tumour growth, along with progressive tissue and organ damage 21. The extreme sensitivity of cardiovascular cells to proteotoxic stress provides a mechanistic explanation for heart disease as the most frequent cause of death from ‘natural causes’ 12. This phenomenon is particularly evident in diseases for which age‐associated physical decline is greatly accelerated—notably Hutchinson–Gilford progeria syndrome (HGPS). HGPS‐afflicted individuals express a defective variant of the nuclear lamin A protein, which rapidly forms toxic aggregates triggering accelerated accumulation of senescent cellular forms in all tissue, promoting inflammatory tissue damage—which is reflected as accelerated physical ageing. HGPS humans typically die at about age 13 from heart failure 22. The primary focus of the present review is an examination of ‘housecleaning’ mechanisms of autophagy, by which cells respond to a major consequence of oxidative damage to cell macromolecules, which is intracellular accumulation of toxic detritus in cells of the heart and other tissue—defined above as proteotoxic stress. Autophagy has recently been recognized as a vitally important cytoprotective process in function of cardiovascular 23 and other tissue 24. The observed protective effects of autophagy are subjected to a significant caveat that autophagic activity may significantly contribute to impaired cellular function 25. Thus, clinical use of strategies that modulate the autophagic response will probably need to be deferred until improved insight is gained into potentially adverse effects of autophagy when applied in the prevention or treatment of disease. This limitation notwithstanding, processes whereby enhancement of autophagy may ameliorate arrhythmogenesis by attenuating the effects of proteotoxic stress provide a paradigm for therapeutic modulation of autophagy in the prevention and management of VF and other arrhythmias. In particular, drug interventions that suppress intracellular accumulation of toxic protein aggregates by suppressing activity of mammalian target of rapamycin (mTOR) and increasing adenosine monophosphate‐activated protein kinase (AMPK) exhibit particular promise—and may have corollary benefit in life extension research, due to inhibition of geroconversion to pro‐inflammatory senescent cell phenotypes, as previously described by the authors 18.

### Autophagy: a major cellular countermeasure to proteotoxic stress

Hypertrophy and senescence of cardiac cells leading to VF and related pathologies may be significantly suppressed by inhibition of mTOR with the drug, rapamycin 26. This effect occurs as a consequence of the normal cellular role of mTOR, which includes constitutive repression of autophagy, best defend as a ‘housekeeping’ mechanism by which a cell clears a wide range of debris, including toxic protein aggregates that promote emergence of senescent pro‐inflammatory cell types. Thus, pharmacological suppression of mTOR, which acts as a negative regulator of autophagy, allows enhanced autophagic clearance of toxic debris produced by high mTOR activity and amelioration of several major pathologies including cardiovascular and neurodegenerative syndromes 18, 27, 28. Autophagy thus appears to have evolved as an endogenous protective countermeasure to proteotoxic stressors and acts primarily to reduce the rate at which hyperinflammatory senescent cellular phenotypes accumulate in tissue 18, 27. These observations notwithstanding, autophagy may exacerbate pathological processes as well as inhibiting disease severity 28, 29. For this reason, it is essential that future strategies for the prevention and management of disease that manipulate autophagic processes be undertaken with an understanding of adverse side effects that may occur as a result of this approach. As the present review is expected to serve as a guide by which improved approaches use autophagy in the prevention and remediation of serious chronic illness, a short but comprehensive description of the molecular mechanisms of autophagy will be considered in the following sections.

## Major modes of autophagy

### Autophagic pathways

**Figure 1:**
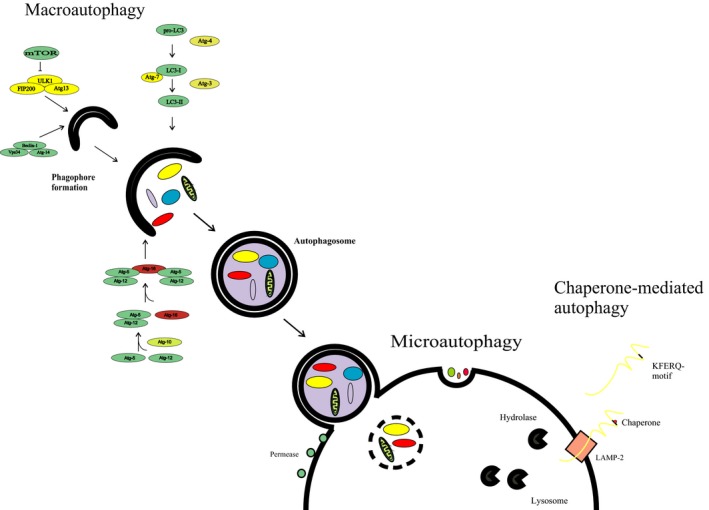
Progression of major macromolecular events in autophagic process. mTORC‐mediated repression of autophagy is relieved by decreased nutrient availability and pro‐autophagic downstream signalling thorough ULK1 and Beclin1–PI3KC3. The lipid mediator PI3P produced by Beclin1–PI3KC3 triggers WIP12/DFCP1‐mediated assembly of the Atg12– Atg5– Atg16 complex, in turn stimulating Atg8 (LC3)‐induced progression of the degradation of targeted materials.

Cells are known to undergo three modes of autophagic activity. Peptides, minor proteins and other small molecules recognized by the cell as potentially toxic are removed by microautophagy, which involves direct uptake by the lysosomes of the targeted material for recycling (Fig. 1) 11. Larger categories of detritus are subjected to degradation and recycling *via* macroautophagy, in which damaged or unusable proteins and cellular organelles become enclosed by double‐walled membrane vesicles called autophagosomes, and then fused with lysosomes for degradation and recycling 30. This process, mediated mainly by ubiquitin and enzyme‐containing complexes called ULK and Beclin1–PI3KC3, is the form of autophagy most frequently utilized by cells for clearance of detritus in a size range capable of severely disrupting cellular homoeostasis 31, 32, 33—and constitutes the form of autophagy most frequently described as a major cellular countermeasure to accumulation of toxic protein aggregates 32, 33. The presence of Beclin‐1 protein (autophagy‐related gene: Atg6) may be used as a reliable biomarker for the occurrence of autophagy in biological samples, including myocardial tissue 34. A third form of the autophagic response is called chaperone‐mediated autophagy (CMA), which is a highly selective quality control programme that degrades nascent polypeptides if they become incorrectly folded 35. During the process, the KFERQ region of the misfolded protein is recognized by chaperons and taken up by the lysosomes *via* channels formed by LAMP‐2 proteins 35. The foregoing descriptions illustrate the role of autophagy in cardiac and other tissue as an adaptive mechanism for maintaining healthy cellular homoeostasis by clearing toxic cellular debris and allowing cells that might otherwise die to survive. Autophagic activity resulting in cell survival appears to be a significant factor in the amount of tissue that may survive for prolonged periods in chronically ischaemic myocardium 36, 37. Moreover, this protective effect may be abolished with autophagy‐inhibiting drugs such as wortmannin 38, 39, 40. These observations notwithstanding, autophagic activity is not always beneficial. Under conditions of extreme tissue stress, high levels of autophagy may initiate cascades of pathological reactions in myocardial cells and, ultimately, deterioration of myocardial function 14.

## Autophagic response hierarchy: cellular housecleaning mechanisms

### Post‐translational quality control and the unfolded protein response (UPR), first‐line countermeasures to toxic detritus

**Figure 2:**
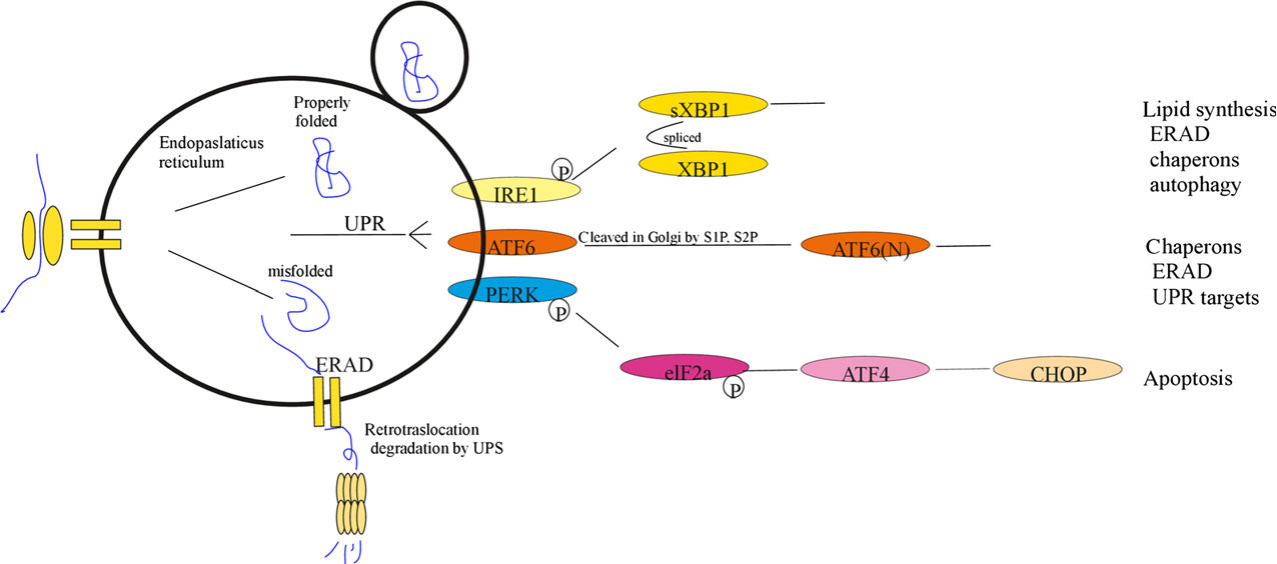
Endoplasmic reticulum‐associated degradation (ERAD) and unfolded protein responses (UPR). Nascent polypeptides emerging into the ER as they are translated from their associated ribosomes undergo folding through linkage of cysteine residues by protein disulphide isomerases (PDIs). N‐terminal glycosylation forms binding sites for calnexin (CNX) and calreticulin (CRT). These chaperones along with Grp78 (BiP) direct misfolded proteins into retrotranslocation out of the ER, into degradation *via* the ubiquitin–proteasome pathway. ERAD constitutes a cell's first‐line countermeasure to the accumulation of potentially harmful metabolic detritus. Misfolded proteins displace GRP78/BiP from PERK, IRE1 and ATF6, thereby activating each factor. The activation of IRE1 mediates XBP1 splicing (sXBP1) and enhances lipids, chaperone ERAD proteins and also promotes autophagy. PERK activation globally reduces protein synthesis *via* eIF2a phosphorylation, and ATF6 is cleaved to nATF6, promoting expression of a variety of UPR target genes.

Autophagy and related quality control mechanisms associated with the endoplasmic reticulum (ER), along with a protein integrity editing process called the UPR, represent a multilayered array of adaptive activities evolved by cells to mitigate debris‐mediated interference with normal cellular function. A cell's first‐line defence against accumulation of potentially harmful debris is a highly selective quality control programme within the ER that degrades improperly folded nascent polypeptides during translation 41. These mechanisms are activated at the earliest stages of post‐translational modification and are relevant to present discussion based on their capacity to promote autophagic activity through triggering of the UPR, a process in which improperly folded proteins that evade immediate post‐translational deletion cause alterations in gene expression that amplify early clearance of misfolded proteins in the ER and may also drive the cell into apoptosis 42. The relationship between UPR activity and autophagy will be discussed further in the next section of the present review. Nascent chains which fail to fold properly despite enzymatic correction become complexed with a 78‐kD chaperone molecule called binding immunoglobulin protein (BiP) also known as glucose‐regulated protein (GRP‐78). Malfolded chains may form aggregates or complex with BiP, to enter a process called endoplasmic reticulum‐associated degradation (ERAD). The process of ERAD quality control involves deposition of misfolded proteins in the cytosol through transient complexes formed with BiP and other chaperone molecules, followed by binding to ubiquitin and degradation *via* the ubiquitin–proteasome pathway 43. A simplified representation of ERAD and related processes is shown in Figure 2. If the burden of misfolded or damaged proteins and toxic complexes formed by these molecules exceeds the processing capacity of ERAD and ubiquitin–proteasome‐mediated degradation, ER stress due to their presence may be alleviated by a cellular adaptive response that augments ER coping mechanisms and may also induce cell cycle arrest or apoptotic deletion, thus eliminating the hazard posed by the cell should its damaged phenotype render it carcinogenic or senescent. These processes, collectively known as the UPR, alter gene expression and cellular metabolism in ways that promote and merge into autophagic degradation of a wide range of cellular detritus 44. UPR activity is represented by three major processes, each defined by outcome of dissociation and subsequent activation of each of the aforementioned BiP/GRP78 ligands. Figure 2 is a schematic representation of the three signalling processes constituting arms of the UPR and major physiological outcome resulting from the activation of each pathway.

### Initiation and preliminary phases of autophagy

Challenges posed to cellular homoeostasis by detritus for which ERAD and UPR responses are insufficiently robust may trigger macroautophagy 31. The first phase of this process, known as autophagic sequestration, involves envelopment of cytoplasmic contents by strongly osmophilic ER‐derived cisternae (vesicles), with membranes biochemically distinct from those of the ER and electron dense, thus allowing these structures to be easily distinguished by electron microscopy 45. Appearance of these primary autophagic vacuoles (AV), called autophagosomes, represents the earliest phase in the evolution of autophagy‐related cellular structures identifiable by microscopic analysis of cellular morphology 45. Autophagosomes fuse with endosomes to form intermediary AV termed amphisomes, which typically contain cytoplasmic material designated for recycling, but retain significant quantities of mostly non‐denatured proteinaceous aggregates in an acidic environment, along with low concentrations of lysosomal enzymes 46. Progression of autophagy continues with enlargement of amphisomes by fusion with autophagosomes, lysosomes and endosomes 46, 47. Eventually, multiple fusions with lysosomes elevate the levels of lysosomal enzymes to facilitate significant degradation of amphisomal contents 48. The aforementioned preliminary stages of the autophagic process occur in tandem with recruitment of the components, which interact to drive degradation of autophagosome contents. These molecules are trafficked to AV membranes beginning in the earliest stages of autophagosome formation and become progressively more co‐ordinated as autophagy proceeds 31.

### Major regulatory and structural components of autophagy

**Figure 3:**
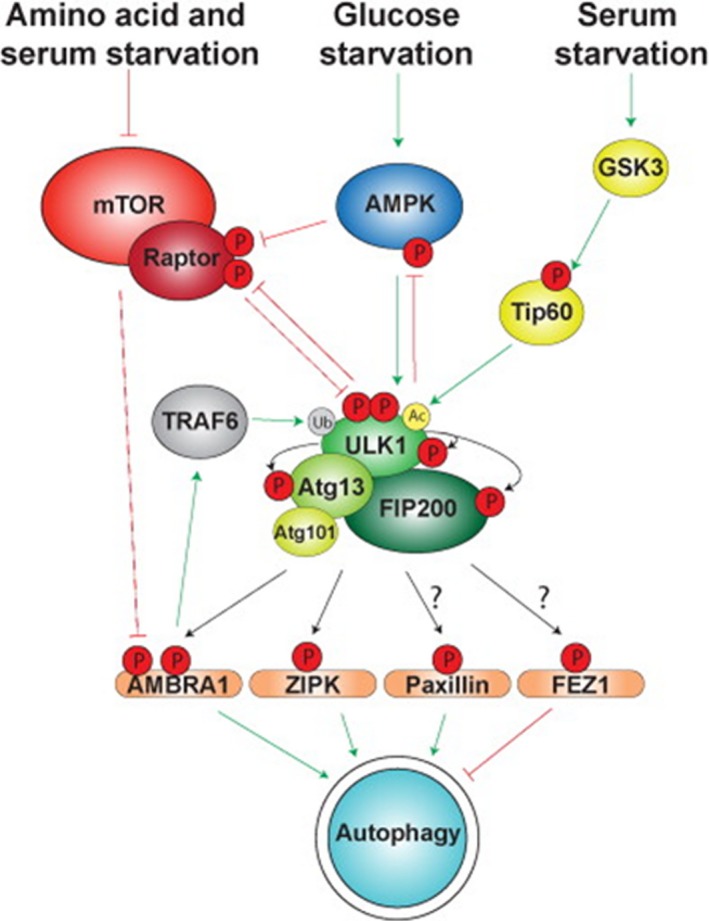
Regulation of and by the ULK1 complex during autophagy activation. Amino acid, glucose and serum starvation activate the ULK1 complex *via* separate signalling pathways. Autophosphorylation of ULK1 may maintain it in a conformation that is favourable for autophagy. Adapted from Wirth M, Joachim J, Tooze S. Autophagosome formation—The role of ULK1 and Beclin1–PI3KC3 complexes in setting the stage. *Seminars in Cancer Biology* 23 (2013) 301–309.

The core machinery of autophagic processes is dependent on 36 Atg proteins, which are highly conserved in evolution 49. Interaction of these components following their assembly into the autophagosomal membranes is tightly regulated by the nutrient‐sensing enzymes mTOR, AMPK and glycogen synthase kinase‐3 (GSK3), the activities of which are modulated by the availability of amino acids, glucose and serum (with component growth factors), respectively. As shown in Figure 3, activities of each of these aforementioned factors promote or repress autophagy through their effect on uncoordinated 51‐like kinase 1 (ULK1), which is a serine/threonine kinase complexed with three major regulatory factors (Atg101, Atg13 and FIP200) 31. The ULK1 complex serves as a major central regulatory element in commitment to undergo autophagy and determination as to whether the outcome will be death or survival of the cell 31.

### Autophagy, mTOR and cellular senescence

Figure 3 shows the functional relationships between the major cellular regulators of autophagy. As described above in section IIa, mTOR is a serine/threonine kinase which, together with its associated regulatory element, raptor, responds to nutrient availability by cell growth‐related synthesis of protein precursors to protein—which, unless counterbalanced with proliferation, results in emergence of swollen and distorted ‘senescent’ phenotypes which express high levels of inflammatory mediators contributing to impairment of tissue and organ function 21. mTOR also acts on ULK1 to repress autophagy 31. The activity of this enzyme is vital for a wide range of activities contributing to healthy homoeostasis by cells, tissues and whole organisms 50; nevertheless, mTOR activity becomes pathological in cells that sustain damage sufficient to activate the p16/p21 tumour suppressor system, causing cell cycle arrest, thus preventing their evolution into cancers, but fails to result in apoptotic or necrotic cell death 18. Cellular senescence may be triggered by damage to the cell as a result of disease or trauma and may also be caused as a result of oxidative stress occurring due to deteriorated genomic organization at the end of an individual cell line's replicative lifespan 18. As described previously in section IIa, senescent cell accumulation constitutes a key contributor to age‐related physical deterioration 18; moreover, deleting these cells as they emerge into senescent phenotypes has been demonstrated to maintain youthful vigour in a transgenic mouse model 51. As nutrient‐driven mTOR activity is a potent negative regulator of autophagy, inhibition of the enzyme with drugs such as rapamycin allows up‐regulation of autophagic processes, resulting in clearance of toxic detritus, which in some cases may restore healthy cellular and tissue function 18. Under physiological conditions, mTOR is a component of one of two complexes, each configured for separate functions in the maintenance of cellular metabolism. These are mTORC1, which includes mTOR and the regulatory proteins Raptor: mLST8 (GL), Pras40, Deptor and Tti1/Tel2. This form regulates mTOR activity based on nutrient energy content and redox status 52. The activity of mTOR in a second regulatory complex, mTORC2, which contains mTOR, along with rapamycin‐insensitive companion of mTOR (RICTOR), mammalian stress‐activated protein kinase interacting protein 1 (mSIN1) and the protein GβL, is also modulated by nutrient availability and composition. The role of mTORC2 in cellular physiology is primarily to mediate changes in cytoskeletal architecture in response to cues from the cellular environment 53.

### Role of ULK1 and Beclin1–PI3KC3 and related proteins in regulation of autophagy

Initiation of autophagy and its progression to either cell death or survival are dependent on feedback interactions between the major nutrient status sensors and ULK as shown in Figure 3. In addition to ULK1, mTOR and GSK3, another complex of autophagy‐associated proteins that plays a critical role in co‐ordinating autophagic activity is Beclin‐1–class III phosphatidylinositol 3‐kinase (PI3KC3) and Atg14L (also known as Barkor), an associated trafficking and catalytic factor 54. Beclin‐1–PI3KC3 responds to interaction between a variety of upstream regulators of autophagy to produce PI3P, which is a lipid mediator responsible for a signal cascade that maintains co‐ordinated progression of autophagy 55. Beclin‐1 thus acts to relay external signals to the cell which trigger autophagy and influence its outcome 55.

### Major macromolecular events in autophagic process

The progression of co‐ordinated interaction between the major protein and vesicular components of autophagy is illustrated briefly in Figure 1. Typically, autophagy may be triggered by the presence of toxic intracellular debris and augmented by a cell's response to nutrient and redox status of its environment 18. The availability to a cell of high nutrient levels stimulates the mTOR–raptor complex to down‐regulate autophagy by direct phosphorylation of the ULK1 enzyme, along with phosphorylation of its associated regulatory element Atg‐13 56. Additionally, the ability of AMPK to promote autophagy in response to decreased nutrient levels or feedback signalling from ULK1 is inhibited by mTORC1‐mediated phosphorylation on Ser757 57, and low nutrient‐mediated decreased mTORC1 activity augments autophagy through de‐repression of pro‐autophagic signalling 31. As shown in Figure 1, signalling through ULK1 and Beclin‐1–PI3KC3 catalyses assembly of Atg proteins into membranes of autophagosomes, initiating the structural progression characteristic of the process. The lipid mediator PI3P produced by Beclin1–PI3KC3 mediates assembly of WIPI proteins, which are structural regulators that facilitate organization of ubiquitin‐like conjugation systems. These systems include the Atg12– Atg5– Atg16 complex and a form of Atg8 (LC3), which undergoes covalent modification by lipidation and subsequently associates with autophagosomes in ways that stabilize the autophagic process. This enhancement results in co‐ordinated expansion and fusion of autophagosomal membranes 58.

## Disruption in autophagic functions: role in disrupted cellular homoeostasis and cardiovascular disease

### Contribution of autophagy and apoptosis to cardiac stress responses

Rapidly emerging insights into mechanisms of autophagy have revealed features of the process, which offer potential to develop enormously valuable approaches for the prevention and management of a diverse range of serious chronic diseases currently refractory to medical intervention. Clinical use of autophagy must nevertheless be undertaken with careful consideration of its damaging capacity for some of the same reasons that induction of apoptosis as part of a therapeutic regimen must be approached with caution. Both apoptosis and autophagy are adaptive mechanisms that have evolved to eliminate damaged cells and cellular material that may pose threats to the health of an organism. Both processes contribute substantially to survival of an individual when occurring at levels sufficient to meet major proximal threats, but become pathological when depletion of functional cells impairs healthy tissue homoeostasis 59. Indeed, studies of *in vivo* responses to haemodynamic stress reported in 2007 demonstrated an inverse relationship between levels of apoptosis and autophagy in hearts of mice responding to cardiac pressure overload 60. Moreover, this balance between the two processes appeared to contribute to the adaptive capacity of hearts of animals subjected to the induced stressors 60.

### Impairment of autophagy degrades cardiomyocyte and heart function

In the aforementioned studies, investigators evaluated contribution of autophagy to induced cardiac overpressure through the use of a strain of mice bearing a transgene for Atg5 (autophagy‐related 5) protein, genetically configured to undergo cardiac‐specific deletion under temporal control in adult mice. As Atg5 is an essential component of autophagy, autophagy in mice bearing this element was abrogated. Adult mice deficient in Atg5 develop hypertrophic hearts with insufficient contractile activity and ventricular dilatation 60. Deficiency in Atg7, another protein that autophagy cannot occur without, has also been observed to disrupt essential homoeostatic processes when experimentally induced in mice 61. Ultrastructural examination of heart tissue from Atg5‐deficient mice revealed cellular changes contributing to malfunction of the organs, including misalignment and aggregation of mitochondria, along with deposition of ubiquitinated proteins and disorganized sarcomere structure 60. Interestingly, these degenerative effects were present concurrent with inactivation of autophagy in hearts of adult mice *via* the inducible Atg transgene, but were delayed by a week following pressure overload when the process was interrupted early in cardiogenesis 60. This outcome suggests that constitutively occurring autophagy is a major mechanism by which size and functional integrity of both cardiomyocytes and whole hearts are maintained within normal parameters 60, 62, 63.

### Autophagy‐related pathophysiology of cardiovascular disease (two major pathological processes)

The major result of dysregulations in autophagy that may cause disease is broadly grouped into two major outcome of impaired autophagic activity. One disease‐associated outcome is failure of autophagosomes to properly mature into endosomal vesicles capable of isolating intracellular detritus. A second class of defect occurs as a consequence of the inability of autolysosomes to effectively degrade the autolysosome contents 64. Cardiac malfunctions arising as a result of each of these two defects are briefly considered below.

### Pathological consequence of autophagic endosomal maturation failure (LAMP2 deficiency)

An example of the first of the two aforementioned categories of autophagy deficiency‐linked cardiovascular disease is observed in mice deficient in lysosome‐associated membrane protein‐2 (LAMP‐2), an extensively glycosylated protein present at high density in lysosomal membranes, which has been demonstrated as essential for normal maturation of autophagic vesicles, in particular proper fusion between early and late endosomes 65. Characterization of the role of LAMP‐2 in constitutive autophagy demonstrated that mice expressing a functionally defective variant of this protein exhibited higher mortality relative to wild‐type mice. Ultrastructural analysis of cardiovascular and other tissues of LAMP‐2‐deficient mice demonstrated extensive accumulation of early autophagic endosomes, along with persistence of long‐lived proteins, which are normally degraded in cells with fully functional autophagic capacity 65. Moreover, cardiac myocytes from LAMP‐2‐deficient animals were eccentric in structure, and contractility of hearts from these animals was significantly impaired 65. Interestingly, humans bearing a deficient gene for LAMP‐2 develop a syndrome called Danon's disease, characterized by disrupted cardiovascular function and skeletal muscle weakness, with numerous features in common with the aforementioned experimentally induced murine variant 66, 67. The parallel emergence of cardiovascular deficiencies in LAMP‐2‐deficient mice and humans, and as a consequence of disabled autophagy, underscores the critical nature of this process to understanding the molecular basis for heart disease and related conditions.

### Pathological consequence of autophagic debris degradation failure (Vici syndrome)

A representative example of failure to fully degrade autolysosome contents is Vici syndrome, a multisystem recessive disorder in humans occurring due to a defect in the gene EPG5 coding for ectopic P‐granules autophagy protein 5, which regulates the ability of late autophagosomic vesicles to degrade their contents 64. Persons afflicted with Vici syndrome experience a cluster of adverse effects, including impaired neurological development immunodeficiency, eye and skin disorders, along with cardiomyopathy 64. Electron and other microscopic examinations of both skeletal and cardiac muscle from persons afflicted with this disorder reveal major cellular myopathic characteristics such as increased numbers of internal nuclei, abnormal glycogen deposits, fibre‐depleted myofibrils, basal lamina redundancy with evidence of exocytosed material between laminar layers, along with morphologically abnormal and maldistributed mitochondria 64. Interestingly, analysis of post‐mortem microscopic cardiac changes in patients with Vici syndrome revealed that the aforementioned histopathological changes were of substantially greater magnitude in left *versus* right ventricle samples 68. This observation underscores the importance of autophagy as a major contributor to cardiovascular health. The left ventricle has evolved to sustain a significantly higher work load than the right, and its component cells are therefore subjected to a higher flux of reactive oxygen species than are tissue of the right ventricle. These in turn likely result in greater accumulation of toxic debris in left ventricular tissue and a greater need for constitutive clearance of this debris to maintain healthy cardiac function. The very high sensitivity of cardiac tissue to the presence of toxic debris 22 makes efficient autophagy essential for the function of left ventricular tissue in particular—and it is therefore unsurprising that patients with Vici syndrome exhibited a greater degree of ultrastructural pathogenic changes in the left *versus* right ventricle.

## Autophagy, diabetic cardiomyopathy and ‘biotherapeutic’ treatment strategies

### Diabetic cardiomyopathy

Diabetic cardiomyopathy (DC) is a major comorbidity of type 2 diabetes mellitus (T2DM), prominently featuring progressive cardiomyocyte loss and accumulation of fibrotic tissue leading to disrupted ventricular function independent of any cardiovascular disease or related condition such as hypertension 69, 70. DC contributes substantially to death and incapacitation among T2DM patients 71; however, at the time of this writing, the pathomechanisms of this disorder have not been characterized in sufficient detail to enable development of effective clinical countermeasures. Insight into DC pathogenesis has recently been provided by studies demonstrating that AMPK activation inhibits DC through restoration of autophagy in cardiac cells, with the concomitant effect of reducing apoptotic depletion of heart tissue 72. These observations, which suggest an inverse relationship between apoptosis and autophagy in diabetic hearts, may be regulated by some of the same underlying molecular interactions that govern occurrence of these two processes in hearts responding to cardiac pressure overload 60. Further investigation of the phenomenon of AMPK‐mediated restoration of autophagy as a countermeasure to DC using a H9c2 cardiac myoblast cell model revealed that elevated glucose decreased AMPK activity, inhibiting Jun NH2‐terminal kinase 1 (JNK1)–B‐cell lymphoma 2 (Bcl‐2) signalling, allowing formation of Bcl‐2–Beclin1 complexes resulting in inhibition of autophagy, whereas low glucose‐activated AMPK with the anti‐diabetic drug metformin promoted JNK1–Bcl‐2 signalling, disrupting Beclin‐1–Bcl‐2 complexes, allowing restoration of cardiac autophagy 72. Similarly, reduced autophagic responses were observed in type I diabetic mice; however, overexpression of Beclin‐1 worsened the diabetes‐induced cardiac injury 73. In a recent study, Kanamori *et al*. 70 have compared the autophagic processes in hearts of type I and type II diabetic animals, using streptozotocin to induce type I DM and db/db mice as type II model. In contrast to the two aforementioned studies, the authors have found decreased Adenosine triphosphate (ATP) content and enhanced AMPK activity accompanied by increased autophagic activity, evidenced by enhanced autophagic protein levels and increased number of AV, in hearts originated from type I diabetic animals. Moreover, inhibition of autophagy further deteriorates normal cardiac functions of the animals, suggesting that autophagy protects type I diabetic hearts. In the same study, decreased ATP content and AMPK activity were also observed. Despite enhanced levels of LC3‐II and SQSTM1 in hearts from db/db animals, microscopic studies revealed reduced numbers of lysosomes and autolysosomes, indicating decreased autophagy. Induction of autophagy by resveratrol restored lysosomal and autophagic activity and improved cardiac functions. Conversely, Munasinghe *et al*. 74 recently demonstrated increased expression of LC3‐II and Beclin‐1 and decreased p62 production, along with increased numbers of autophagosomes in samples taken from right atria of diabetic patients, in comparison with non‐diabetic control donors. The role of autophagy in diabetic heart appears mostly protective, but remains to be comprehensively characterized separately for type I *versus* type II diabetic conditions as the aetiology of the two variants of diabetes is different, for example lack of insulin in type I and insulin resistance in type II diabetes, as insulin itself regulates autophagy *via* mTOR 70, 75.

Recent studies have demonstrated that autophagy may be induced at levels strongly protective against myocyte depletion and cardiac functional derangements associated with DC by haem oxygenase‐1 (HO‐1), a main component of cellular redox regulation and antioxidant defence 76. Related investigations by Mahmoud *et al*. 77 have further shown that phytochemical induction of HO‐1 derived from sour cherry seed suppresses disease‐associated inflammatory signalling in cells from patients with T2DM, rheumatoid arthritis, which is also a major comorbidity of cardiovascular disease 78. These investigators further demonstrated that a topically applied phytochemical inducer of HO‐1 derived from flavonoid sour cherry seed extract (SCSE) dramatically ameliorated the major symptoms of osteoarthritis (OA) in human patients, by targeted inhibition of inflammatory cytokine expression by CD3+ T cells 79. This research initiative was the first successful demonstration of a ‘biotherapeutic’ treatment of OA. Biotherapeutic methods attack root causes of disease (in this case, CD3+ T cell production of inflammatory cytokines), rather than simply treating symptoms. Conversely, most currently available ‘orthodox’ treatments for OA and all other inflammatory syndromes rely on palliative methods, typically small‐molecule drugs such as corticosteroids that are only temporarily effective and severely debilitating with sustained use. In the above example, the phytochemical HO‐1 inducer utilized completely avoided adverse side effects.

### Autophagic influence on cell survival: clinical relevance

Ongoing studies of ischaemic disease in cardiovascular tissue reveal that autophagic activity resulting in cell survival appears to be a significant factor in the amount of tissue that may survive for prolonged periods in chronically ischaemic myocardium 36. Moreover, this protective effect may be abolished with autophagy‐inhibiting drugs such as wortmannin, a pharmacological inhibitor of autophagy 40, 80. An autophagic process specific for clearance of defective mitochondria called ‘mitophagy’ has also been shown to protect against ischaemia–reperfusion (IR) injury 81, 82. This outcome notwithstanding, precautions to prevent autophagic damage to the heart must be a major hallmark of clinical development of autophagy‐based methodologies in cardiovascular medicine as under conditions of extreme tissue stress, high levels of autophagy may initiate cascade pathological reactions in myocardial cells and, ultimately, myocardial function derangement 14.

### Evaluations of autophagy as a clinical correlate and possible contributor to atrial and ventricular fibrillation

Recently, authors of the present report evaluated the correlation between IR‐induced VF and autophagy in heart tissue. In this investigation, it was observed that myocardial muscle of ischaemia/reperfusion (I/R)‐injured isolated fibrillated mouse hearts expressed significantly higher level of Beclin‐1, LC3B‐II and the LC3B‐II/LC3B‐I ratio than non‐fibrillated hearts 83. These results establish a functional correlation between VF and autophagy; however, cause–effect relationships between the two phenomena remain to be defined. The present study and ongoing investigations are designed to include components that will improve insight into the mechanistic relationships between VF and autophagy. Although correlation between VF and ischaemia/reperfusion injury has been extensively described, evidence linking autophagy to VF is limited at the time of this writing to the pioneering paper by authors of this report 83 and two others, in which the interaction of the two pathways is discussed indirectly 84, 85. The authors acknowledge that research in this topic area is not sufficiently mature at this point to comment extensively on it and include mention of possible relationship between VF and autophagy to provide a perspective on promising research directions.

**Figure 4:**
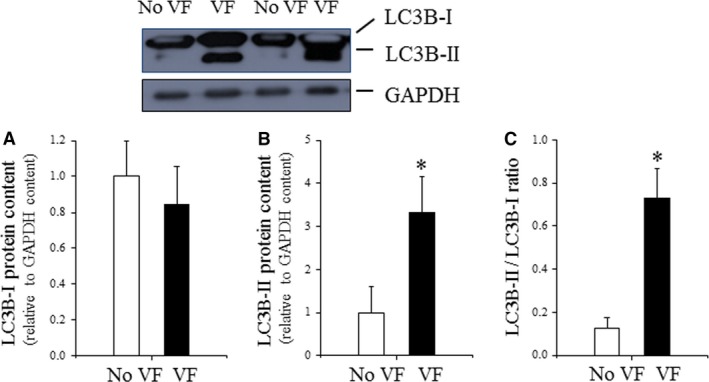
LC3B content in VF and non‐VF post‐IR heart tissue. (**A**) Cardiac LC3B‐I expression evaluated by Western immunoblotting and expressed relative to GAPDH content; (**B**) cardiac LC3B‐I expression evaluated by Western immunoblotting and expressed relative to GAPDH content. (C) Cardiac LC3B‐II/LC3B‐I ratio evaluated by Western immunoblotting (data are presented as n = 4 per group, *P < 0.05 No VF *versus* VF hearts.) Adapted from Meyer G, Czompa A, Reboul C, Csepanyi E, Czegledi A, Bak I, Balla G, Balla J, Tosaki A, Lekli I. The cellular autophagy markers Beclin‐1 and LC3B‐II are increased during reperfusion in fibrillated mouse hearts. *Curr Pharm Des*. 2013; 19 (39): 6912–8.

Some insight provided by the authors is provided in Figure 4, which illustrates the strikingly elevated levels of LC3B‐II and the LC3B‐II/LC3B‐I ratio in muscle from hearts undergoing VF, *versus* non‐VF hearts. Recently, Yuan and colleagues 82 have reported an enhanced level of p‐AMPK in atrial tissue obtained from patient suffering from chronic atrial fibrillation (AF). Furthermore, the level of LC3BII was also enhanced in AF patient, indicating an AMPK‐dependent autophagic activation. Similarly, time and AMPK‐dependent autophagy were found in canine model of AF 86. In another study, enhanced autophagy was found in atria originated from patient suffering from mitral regurgitation in the presence or absence of AF 87. However, in the aforementioned studies, the functionality of autophagic process was not studied. Earlier, Garcia and colleagues have found enhanced number of AV in atrial tissues from patient exhibiting postoperative AF 88. However, in this study, the level of LC3II was decreased compared to non‐fibrillated atrial tissue, indicating an impaired autophagic process in the atrial tissue. The connection between ventricular arrhythmias and autophagy is further supported by a recent study conducted by Lu *et al*. 84. After cardiac arrest induced by VF, resuscitated animals received epinephrine in the absence or presence of esmolol. Animal that received epinephrine and esmolol simultaneously exhibited lower level of Beclin‐1, and Parkin indicated that esmolol may reduce overactivation of autophagy and mitophagy 84. Taken together, the role of autophagy in myocardial arrhythmias is still unclear and further studies are required.

## Autophagy‐based cardiovascular therapies

### Approaches for therapeutic utilization of autophagy: mechanistic considerations

Strategies for the prevention and management of cardiovascular pathologies that modulate autophagic activity offer several major advantages over currently available therapies. One major benefit to patients of these approaches is that autophagy may often augmented using combinations of dietary phytochemicals 38, exercise 39 and various other approaches that are non‐toxic and non‐invasive. Another significant advantage to development of autophagy‐based clinical methods is the potential for the effective management of several very serious disorders that are refractory to currently available therapies. For example, although approximately 50% of the injury sustained by human hearts as a result of cardiac infarction is due to I/R injury 89, there are no currently available preventive or therapeutic countermeasures to I/R‐mediated tissue damage. Encouragingly, however, recently 90 reported that an inhibitor of histone deacetylase (HDAC), suberoylanilide hydroxamic acid (SAHA), increased cardiac autophagy in an animal model at sufficiently robust levels to reduce myocardial infarct size in I/R‐injured hearts.

### Clinical modulation of autophagy by calcineurin‐inhibitory drugs

Another very exciting event in the exploration of autophagy as a cardiac therapy tool is a report by He *et al*. 91 that suppression of cardiac autophagy with calcineurin enhances oxidative damage to cardiomyocytes as a result of calcineurin‐mediated inhibition of AMPK. The aforementioned study additionally demonstrated that AMPK‐mediated autophagy could be restored using FK506, an immunosuppressant drug widely used following organ transplant surgery to suppress allograft rejection. This development is of particular significance in the context of findings by Haines *et al*., 1 demonstrating that FK506 effective dosage and thus toxicity could be drastically reduced by co‐administration with a phytochemical mixture that reduced calcium influx to cells of the heart, thus enabling very low dosages of the drug to be used with high pharmacological effectiveness. The capacity to use these and related strategies in ways that drastically reduce cardiotoxicity of drugs used to prevent or treat heart failure opens up an expanding range of opportunities for improvements in cardiovascular medicine. A clinical venue, in which autophagy‐related methods hold particular promise, is in the rapidly expanding field of stem cell therapy. Approaches to optimizing clinical benefits in this very exciting field are described below.

### Strategies for therapeutic use of the unfolded protein response (UPR)

The UPR, post‐translational quality control countermeasure to the effects of proteotoxic stress, described in section IVa of this article, offers expanded strategies for targeting molecular mechanisms leading to cellular senescence, along with a plethora of pathological outcome, including arrhythmias. For example, studies of the effects of oxidative stress on mitogen‐activated protein kinase (MAPK) signalling and UPR induction in a neuronal‐like catecholaminergic PC12 cell model reveal that phosphorylation of eIF2α by PERK is observed to up‐regulate expression of haem oxygenase‐1 **(**HO‐1), through ATF4‐mediated activation of the Nrf2 pathway, which controls several genes critical for antioxidant defence, including HO‐1 92. HO‐1 expression is a major cellular countermeasure to oxidative stress. Inducers of its activity are emerging as a therapeutic tool for the prevention and management of a wide range of diseases 93, and it has recently been shown to counteract the effects of DC in mice through enhancement of autophagy in heart tissue 76. This aforementioned dichotomy of cellular response to PERK activation underscores a fundamental flaw of the UPR with respect to its usefulness to an organism as an adaptive mechanism to ER stressors. Although it has evolved as a highly conserved adaptive reaction to toxic effects caused by accumulation of misfolded proteins, activity of the UPR in response to protein aggregates may itself cause tissue damage in response to heavy burdens of such detritus, which typically occur as main features of several serious chronic neurological disorders, notably Parkinson's disease, Huntington's disease, the prion diseases and Alzheimer's disease 44. Constitutive UPR activation which develops to alleviate ER stress in these syndromes has pathological consequences that in the balance render activation of this pathway, a significant contributor to their pathogenesis 44. For instance, the severity of prion disease in mice was observed to strongly correlate with progressively increasing EIF2α phosphorylation, with resultant suppression of translation and reduced availability of key synaptic proteins, neuronal cell death and debilitation of affected animals 94. Significantly, oral treatment of mice with a PERK inhibitor, which curtailed its phosphorylation of EIF2α, allowed resumption of protein translation to levels sufficient to restore normal neurological function and abrogate progression of the disease 94. Future therapeutic use of PERK inhibitors may enhance the overall effectiveness of UPR‐mediated alleviation of aggregate‐mediated cellular stress; however, as selective inhibition of PERK nevertheless allows the IRE1‐ and ATF6‐mediated arms of the UPR (shown in Fig. 2) to retain functional activity, the benefits of curtailing EIF2α phosphorylation are likely to outweigh the adverse effects. Ongoing drug discovery investigations will reveal to what extent this is true.

## Autophagy and telocyte networks

For approximately 10 years prior to the date of this writing, emerging insight into the ultrastructure of cardiovascular tissue and mechanisms by which its component cells interact to maintain healthy homoeostasis have offered exciting promise for the prevention and remediation of arrhythmic disorders, by enhancement of cardiac regenerative potential. A particularly strong contributor to these advances has been a rapidly evolving characterization of telocytes, a cell type first described in 2010 by a research group led by Professor Laurențiu M. Popescu. Subsequent exploration of telocyte biology revealed that these cells exert subtle but powerful effects on a wide range of tissues, including an intriguing capacity to stabilize the stem cell niche 95. This last feature is particularly significant in the context of current efforts to restore healthy function of the heart and other organs by engraftment of progenitor stem cells derived from various tissues, including umbilical cord blood, adipose and hematopoietic tissue, embryonic stem cells, multipotent mesenchymal stromal cells and other sources. At the time of this writing, there is no evidence that telocytes are involved in VF; moreover, their role in autophagy is in the preliminary stages of definition as described by the authors in Haines *et al*. 18, 95. A comprehensive analysis of how these cells might affect VF awaits the outcome of ongoing investigations in progress. This notwithstanding, it was observed in rat model investigations that tissue cardiac telocytes were depleted during myocardial infarction and improved resistance to and recovery from ischaemic injury 96 and that intramyocardial transplantation of cardiac telocytes decreased myocardial infarction and improved post‐infarcted cardiac function 97. The significance of these findings to possible contribution of telocyte function to arrhythmias is that treatments capable of inhibiting ischaemia/reperfusion‐mediated infarct zone extent (in rat heart) also improve cardiac function and delay post‐ischaemic onset of VF 98. These indirect findings not confirm telocyte involvement in arrhythmogenesis, but suggest that ongoing studies may reveal therapeutic targets involving telocyte function that allow improvement of VF and related cardiovascular syndromes, through modulation of autophagic processes and resulting amelioration of proteotoxic stress.

## Conclusions

As described in the foregoing sections, autophagy is a potent adaptive mechanism for maintaining healthy cellular homoeostasis by clearing toxic cellular debris and allowing cells that might otherwise die to survive in functional forms that do not pose a hazard to surrounding tissue (as do senescent and carcinogenic forms). It is anticipated that understanding of autophagic processes will be particularly valuable in the maintenance of cardiac health, with improvements in quality of life for elderly persons, being a major outcome. This prediction is nevertheless made with the caveat that autophagy has the capacity to become pathological and development of therapies based on its induction must be undertaken with caution. A common feature of the various physiological processes to which autophagic activity is a contributor is that many stressors, in particular the toxic by‐products of oxidative metabolism, accumulate in progressively larger numbers of cells over time, resulting in loss of functional tissue through cell death, cancers and emergence of senescent cellular phenotypes that damage tissue through expression of inflammatory mediators. Indeed, the progressively pro‐inflammatory tissue environment and decline of regenerative potential and tissue viability define physical ageing on a very basic level 18. Autophagy may be viewed as a ‘braking system’ which works against age‐ or trauma‐associated physical decline, but is ultimately futile as individual tissues of an individual are depleted of functional quiescent cells and progenitor stem cells and are overwhelmed by the pro‐inflammatory effect of damaged cells 18. A fascinating implication of this phenomenon is explored by Herman Pontzer *et al*. 99. These investigators demonstrated that within given time periods, the total energy expended by primates is much lower than placental mammals 99. The significance of this observation in the context of tissue‐deteriorating levels of proteotoxic stress is that autophagy in primates is likely to be more effective in holding off ‘wear‐and‐tear’‐related (*i.e*. pro‐inflammatory) physical decline in primates than in shorter‐lived animals. This hypothesis is currently speculative and unsupported by direct proof, but certainly defines a future direction in autophagy‐related research.

## Conflict of interest

The authors confirm that there are no conflict of interests with respect to any of the topic material presented herein.
